# Ultrasound-Guided Fine Needle Aspiration of Deep Thyroid Nodule: Is There a Correlation between the Nodule's Depth and Nondiagnostic Results?

**DOI:** 10.1155/2022/8212636

**Published:** 2022-08-29

**Authors:** Majd Asakly, Raed Farhat, Nidal El Khatib, Ashraf Khater, Alaa Safia, Marwan karam, Saqer Massoud, Taiser Bishara, Yaniv Avraham, Adi Sharabi-Nov, Shlomo Merchavy

**Affiliations:** ^1^Otolaryngology, Head & Neck Surgery Unit, Rebecca Ziv Medical Center, Safed, Israel; ^2^Bar-Ilan University's Azrieli Faculty of Medicine, Safed, Israel; ^3^Research Wing, Rebecca Ziv Medical Center, Safed, Israel

## Abstract

**Objective:**

To evaluate whether thyroid nodule depth correlates with nondiagnostic results in ultrasound-guided fine needle aspiration cytopathology.

**Background:**

Many factors correlate with nondiagnostic ultrasound-guided fine needle aspiration cytology (FNAC) results, including older age, macrocalcification, small-sized nodules, aspirin medication, and cystic portion in more than 50% of the thyroid nodules. However, there are few studies which have examined whether there is a relationship between the depth of nodules and the percentage of nondiagnostic results in cytology (Bethesda category I). We conducted this study in order to investigate if such a correlation exists.

**Materials and Methods:**

FNAC was performed on 283 thyroid nodules between January 2019 and December 2020. Cytological analyses of the nodules were reviewed and sorted as nondiagnostic and diagnostic according to the Bethesda score. Patient files and ultra sound (US) scans were reviewed for clinical information (such as age, sex, and ethnic group) and sonographic features of nodules (such as depth, size, cystic portion, type of calcification, and echogenicity) and were compared between the nondiagnostic and diagnostic nodule results. The depth of a nodule was calculated as the shortest distance from the skin to the most superficial border of the nodule in the axial plane, using our medical center's computer program, which allows reviewing all saved shots of the US scan.

**Results:**

Age, sex, and ethnicity were not significantly different between the nondiagnostic group and the diagnostic group (*p* > 0.05). Nodule diameter, cystic portion, calcification, and echogenicity were also not associated with the frequency of nondiagnostic results. The depth of nodules ≥9 mm was correlated with nondiagnostic US-guided FNA cytological results (OR = 2.55, *p*=0.018).

**Conclusions:**

Deep thyroid nodules correlated with nondiagnostic US-guided FNA cytological results. Further studies are needed for optimizing the approach to deep thyroid nodules in order to improve the efficacy of FNA in deep thyroid nodules.

## 1. Introduction

Thyroid nodules are common, being diagnosed in 34% (27% in men, 41.7% in women) of the adult population [1–3]. The majority of nodules are benign [4]. Ultrasonography (US) is the most important diagnostic tool in the workup of thyroid nodules. The clinical importance of thyroid nodule diagnosis rests on the need to exclude thyroid cancer, which occurs in 5%–15% of cases in correspondence to age, sex, previous exposure to ionizing radiation, family history, and other factors [4–8].

In the last decades, the US-guided fine needle aspiration cytology (FNAC) diagnostic method has become the gold standard for cytopathology differentiation between benign and malignant nodules [9, 10]. US-guided FNAC usually reaches high rates of sensitivity, specificity, and cell adequacy, ranging from 57 99%, 45–99%, and 90–97%, respectively [7, 11–13]. US-guided FNAC has therefore reduced the rate of unnecessary thyroidectomy [14].

The Bethesda system for reporting thyroid cytopathology (TBSRTC) standardized the reporting of thyroid cytopathology results [15]. It includes six diagnostic categories, which are linked to certain ranges of malignancy risk and clinical management guidelines [16]. In TBSRTC, inadequate samples are reported as Category I. This category applies to samples that are nondiagnostic or unsatisfactory due to obscuring blood, overly thick smears, air drying of alcohol-fixed smears, or an inadequate number of follicular cells that got on the aspirates [17].

A high nondiagnostic rate is correlated with older age, macrocalcification, small-sized nodules, cystic portion more than 50% of the thyroid nodules, hypoechogenicity, heterogeneous echogenicity, aspirin medication, lesser experience of the performing physician, lower procedural volume, and aspiration without rapid on-site evaluation (ROSE) [14, 18, 19].

High nondiagnostic FNAC result rates continue to hinder the full potential of FNAC as a diagnostic tool. The percentages for nondiagnostic US-guided FNAC in the literature range widely from 2 to 29% [16, 18, 20, 21]. Such percentages of nondiagnostic results increase the length of time to definitive diagnosis, patient stress, costs, and lead to unnecessary thyroidectomy. Moreover, studies have shown that nondiagnostic samples can harbor malignancy in 7% to 28% of cases [7, 18, 20].

Based on our experience in examining deep nodules, needle navigation and nodule aspiration may be more challenging. Surprisingly, we found only two studies investigating the relationship between the depth of thyroid nodules and the rate of nondiagnostic FNA cytology results [19, 22].

In this study, we aim to evaluate whether thyroid nodule depth as measured by US can predict nondiagnostic cytological results in thyroid FNAC.

## 2. Materials and Methods

### 2.1. Study Population

A retrospective chart and image review was conducted on 215 patients with 283 thyroid nodules who underwent US FNAC between January 2019 to December 2020 in Ziv Medical Center, Israel. The local Ethics Committee approved the protocol. Clinical information, US, and FNA cytology records were reviewed for all patients.

We followed the National Comprehensive Cancer Network (NCCN) and the American Thyroid Association (ATA) guidelines for deciding which nodules were candidates for sampling. High risk features considered included undefined or lobulated margins, microcalcifications, taller-than-wide shape, hypoechogenicity, extrathyroidal extension of thyroid nodule, and prior exposure to radiation [23, 24].

### 2.2. Ultrasound Examination

**Figure 1:**
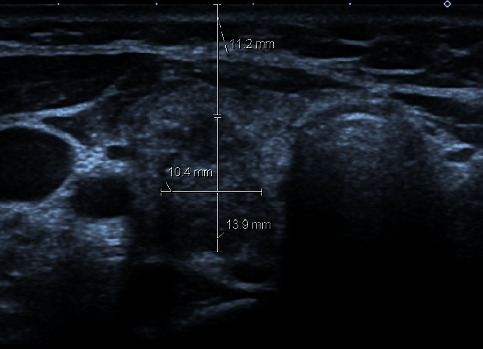
Nodule depth (11.2 mm), and two diameters of the nodule; dimension A (10.4 mm) and dimension B (13.9 mm).

A single head and neck surgeon with more than 10-years' experience in the area of thyroid malignancy performed all the US-guided FNA procedures. The depth of each nodule was considered as the shortest distance from the skin to the most superficial margin of the nodule in the axial plane (Figure 1). Every evaluated nodule was assessed for size, cystic component, calcification, location of nodule, and echogenicity. The size of the nodule was assessed by measuring two diameters: a horizontal diameter (dimension A) and an anterior-posterior diameter (dimension B) (Figure 1). Nodules without the cystic component were considered as solid, otherwise the cystic portion was classified as less than 50% or more than 50%. We excluded all nodules that were purely cystic, because these nodules are invariably nondiagnostic. Calcification when it existed was classified as microcalcification or macrocalcification, when calcification foci size was less than 1 mm or bigger than 1 mm, respectively. Echogencity of a nodule was classified as hyperechoic, isoechoic, hypoechoic, and heteroechoic. Location of the nodule was classified as in the right lobe of the thyroid gland, left lobe, or into the isthmus.

### 2.3. Ultrasound-Guided FNA Procedure

All biopsies were performed using an A 23 Gauge needle, attached to a 3 ml syringe. The needle was 30 millimeter long, not measuring the hub. When it was attached to the syringe, their length together was 130 millimeters. The surgeon adjusted the ultrasound depth and focus as needed to get better visualization of the nodule and the needle tip.

Under US guidance, we collected three syringes (passes) from every nodule. The skin overlaying the nodule was locally anesthetized by lidocaine + prilocaine 2.5% + 2.5% cream (Rafarm S.A, Athenes, Greece) which was applied for 45 minutes for minimum, and the skin cleaned and sterilized with alcohol 70% solution (Floris, Israel). The needle was penetrated into the nodule by holding the syringe, and without the use of a holder. It was moved back and forth within the nodule. An equal vacuum was created on the syringe from the moment the tip of the needle was inserted into the nodule until the hub of the needle was filled with aspirated material and then it was extruded from it.

### 2.4. Cytological Preparation and Diagnosis

Following every FNAC procedure, an experienced cytopathologist immediately expelled the collected materials on glass slides, smeared, and directly fixed the specimen in 95% ethyl alcohol. Staining was performed using the Papanicolaou method. ROSE was not performed. The samples were reported using the Bethesda System. An adequate sample was defined as having at least six clusters of thyroid follicular cells (10 cells) for each specimen. When fewer cells were obtained but abundant colloid was observed, the specimen was considered adequate and benign. Whenever an atypical or other diagnosis (such as thyroiditis) was detected, the specimen was considered as adequate. The chief cytopathologist reviewed and confirmed the results of all slides.

### 2.5. Statistical Analysis

Quantitative data were shown as mean ± standard deviation, whereas numbers and percentage were provided for the qualitative data. Pearson's chi-square test was used to examine the relationships between study groups for categorical parameters such as noudel depth, time of surveillance, age, and both A and B dimensions. Variables were tested for normality according to Kolmogorov–Smirnov. After that, we applied the ROC curve constructed, the chi-square test to measure the differences between the study groups (Bethesda 1 vs. Bethesda 2–6), and the Kruskal Wallis H test to measure the differences between all of the study groups. Multiple logistic regression assessed the correlations between the study groups and risk factors, providing ORs and 95% confidence interval (CI), with adjustment for significant and potential (age and gender) confounders.

A *p*-value of 5% or less was considered statistically significant. The statistical analyses were analyzed using the SPSS version 25 (SPSS Inc., Chicago, ILL, USA).

## 3. Results

A total of 283 nodules were biopsied from 215 patients. Of these, 40 aspirations (14%) were classified as nondiagnostic. The other 243 aspirations (86%) were diagnostic and were categorized as follows: 227 as benign, 6 as atypical cells or follicular lesions of undetermined significance, 3 as follicular neoplasm or suspicious for follicular neoplasm, 2 as suspicious for malignant, and 5 as malignant.

The average age was 57.5 ± 14.8 (Mean ± SD), and 175 (82%) of the patients were females. Baseline demographic data of patients with nondiagnostic nodules were not different compared with the diagnostic group (Table 1).

Kolmogorov–Smirnov test of normality indicated that nodule depth, time of surveillance, age, and both A and B dimension variables did not follow a normally distributed shape. Thus, we applied Mann–Whitney nonparametric tests to measure the differences between study groups (Bethesda 1 vs. Bethesda 2–6). By evaluation of sonographic characteristics, the portion of nondiagnostic specimens increased with deeper location (12.1 ± 4.7 mm in the Bethesda 1 group vs 9.7 ± 6.8 mm in the Bethesda 2–6 group, *p* < 0.001)) and shorter diameters of nodules (Dimension A: 14.5 ± 5.3 mm in the Bethesda 1 group vs 17.2 ± 8.3 mm in the Bethesda 2–6 group, *p* < 0.05 and Dimension B: 13.5 ± 6.7 mm in the Bethesda 1 group vs 16.4 ± 7.8 mm in the Bethesda 2–6 group, *p* < 0.01). There was no significant difference in correlation to the side of the nodule, cystic portion, calcification, or type of echogenicity.

Using the ROC curve constructed did not yield a significant result according to the AUC (*p*=0.690). Therefore, we used the chi-square correlations in order to assess the correlation between the Bethesda groups (1 vs. 2–6) and the depths of the nodules. By using the chi-square test (Table 2), we found that a depth of 8 millimeters is a clear cut-off which can predict nondiagnostic FNA cytology (Table 2). We used the binary logistic regression analysis (Table 3) for determining if there are multifocal influences on nondiagnostic specimens. For this purpose, we chose variables (from Table 1) with *p*- value less than 0.05. The nodule's depth and diameter were the only candidate characteristics. In the multivariate analysis, nodules with a depth of ≥9 mm significantly increased the odds of nondiagnostic FNA (OR, 2.5; *p*=0.022; CI, 1.14–5.45), when compared with the depth <9 mm (Tables 2 and 3). Nodule diameters were not significantly different between the two study groups after cancellation of confounders. Kruskal–Wallis nonparametric test was performed to examine the difference in the nodule depth between the different Bethesda groups [1–6]. The only significant difference was found between groups 1 and 2 (*p* < 0.01). No differences were found between the Bethesda 1 and any of the Bethesda 3–6 groups.

## 4. Discussion

Due to its high sensitivity and specificity, U- guided FNA is the gold standard for thyroid nodule evaluation. The number of ultrasound-guided FNAs has risen.

Over the last two decades, the number of ultrasound-guided FNAs has increased dramatically due to increased acceptance of the additional benefit of ultrasound guidance [10, 19]. Still, nondiagnostic FNAC result rates continue to hinder the full potential of FNAC as a diagnostic tool [18, 25].

Nondiagnostic results might be related to operator skills and expertise of the cytopathologist [5–8]. Therefore, to avoid bias, all thyroid nodules included in our study underwent US-guided FNA by the same experienced operator and all slides were interpreted by the same experienced cytopathologist.

In our study, we found that the depth of thyroid nodules correlates independently with nondiagnostic FNA cytology results. A cut-off of 9 mm depth increased the odds of nondiagnostic FNA results. The clinical characteristics of the patients were not significantly different between the diagnostic and nondiagnostic group, and they were not shown to be correlated with a nondiagnostic result of the FNA.

There is a paucity of research regarding thyroid nodule depth, especially its relationship with nondiagnostic results. Xia et al. also found a correlation between nodule's depth and accuracy of the exam; in their research, they found that nodule depth of 15 mm or more was an independent factor for nondiagnostic results [22]. Kavanagh et al. also found that the average depth of nondiagnostic thyroid nodules was 15 mm, and it was significantly deeper than the diagnostic group. [19] Our study and both of those studies considered the depth of a nodule as the shortest distance between the skin and the outermost margin of a nodule. Both studies found no relationship between nodule size (as opposed to depth) and nondiagnostic FNA cytology results.

The tendency of FNAC of deep nodules to be nondiagnostic may result from a few technical entities: difficulty to identify properly the nodule in the thyroid gland by the US device, difficulty in navigating the needle's tip to the nodule through the soft tissue of the neck, and the difficulty to aspirate the nodule. More than that, aspirating a deeper nodule is followed by greater pain and discomfort to the patient, which makes the examination even more difficult due to the patient's movements. Therefore, deeper nodules may take more time to aspirate. This issue is of outmost importance, because the more the needle stayed into a nodule, more blood could enter the aspirate and obscure the cytological material which hinders the cytological examination and may lead to nondiagnostic cytological results.

One study showed that hypoechoic and hyterogenous nodules are prone to be less diagnostic compared with isoechoic or hyperechoic nodules [26, 27]. This association was theoretically related to fibrosis, hemorrhage, necrosis, and high cellular structures in these nodules. In another study, the highest diagnostic rates were in the hypoechoic nodules and lowest rate in heterogenic nodules[28]. We did not find correlation between echogenicity of thyroid nodule and the nondiagnostic rate of FNA cytology.

Other factors that might influence the nondiagnostic rate but were not examined in this study might be the needle width and the use of the French technique. There is some evidence that on the one hand, wide core needles associate with more cellular aspirates, but on the other hand, they are associated with more obscuring red blood cells; therefore, they are the same diagnostic as narrow core needles [26, 27, 29]. There is some evidence that nonaspiration fine needle cytology, or “the French technique” reduces the number of obscuring blood and has a better sampling accuracy [30–32]. In this technique, a free needle with an uncovered hub is inserted back and forth into the thyroid nodule without activating negative pressure. However, other studies have failed to show superiority of nonaspiration cytology over aspiration cytology [33–35]. In the case of deep thyroid nodules, using a mere needle by holding it into the hub could be manually challenging. Therefore, an alternative option would be to connect the needle to a syringe in which the plunger has been removed. This complies with the rule that the needle's hub should be exposed to the outer atmosphere, and we are able to better control the needle by holding the syringe. This technique may improve the navigation of the needle's tip into the thyroid nodule.

The percentage of nondiagnostic FNA cytology in our study was 14.1% (40/283). This is compatible with studies from other centers [16, 19, 22, 25, 36].As mentioned previously, the range of nondiagnostic FNA cytology is wide, and we believe that this variability relates to the difference in the nodule characteristics associated with nondiagnostic cytology between different study groups, physician experience, aspiration technique (short vs long axis), and variability in the use of ROSE. ROSE is a microscopic evaluation of the cytological material immediately after the aspiration. There is controversial evidence about the utility of using a ROSE for improving the diagnostic rate of US-guided FNA of thyroid nodules [37]. Some studies show a significant impact of ROSE on the nondiagnostic rate of FNA of thyroid nodules [38, 39]. Even in a case where a relatively low initial rate of nondiagnostic results has been observed using ROSE could have significantly improved the accuracy of FNA cytology [18]. ROSE could be efficient on subcentimeter, mixed solid-cystic, macrocalcified, and hypervascular nodules [40].On the other hand, one meta-analysis concluded that the utility of ROSE depends robustly on the initial adequacy rates, the higher the initial rate of nondiagnostic FNA cytology, the bigger the impact of ROSE and vice versa [41]. Generally, ROSE requires extended duration of the procedure, longer patient discomfort, and higher costs [38]; however, it may have a significant implication on the subgroup of deep nodules.

Our study is based on large samples of patients, with a wide diversity of demographic and clinical characteristics that were included in the analysis.

There were some limitations to our study. One limitation is that this was a retrospective study, so a selection bias could not be avoided. Another limitation is that ROSE was not applied on the study's population.

## 5. Conclusion

In our study, we showed that deeper thyroid nodules are associated with a higher rate of nondiagnostic FNA cytology. Different FNA methods may improve the diagnostic rate of deep thyroid nodules, reduce recurrent aspiration, and minimize patient discomfort. Further studies are needed for optimizing the approach to deep thyroid nodules in order to improve the efficacy of FNA in deep thyroid nodules. These future studies could also focus on the length and gauge of the needles, as well as the duration of the FNA procedure.

## Figures and Tables

**Table 1:** Demographic and ultrasound clinical characteristics of the study groups.

Variables	All nodules (*n* = 283)	Bethesda 1 (*n* = 40)	Bethesda 2–6 (*n* = 243)	*p*-value
Gender, female (*n*, %)	234, 82.7	34, 85.0	200, 82.3	0.676
Age, years (mean ± SD)	57.5 ± 14.8	57.3 ± 14.9	57.5 ± 14.9	0.817

Ethnicity (*n*, %)				
Jewish	193, 68.2	27, 67.5	166, 68.3	0.919
Arab	90, 31.8	13, 32.5	77, 31.7	

Side (*n*, %)				
Right	156, 55.2	26, 65.0	130, 53.5	0.176
Left	113, 39.9	11, 27.5	102, 42.0	
Isthmus	14, 14.9	3, 7.5	11, 4.5	
Diameter A (horizontal), mm (mean ± SD)	16.8 ± 8.0	14.5 ± 5.3	17.2 ± 8.3	0.038
Diameter B, (anterior-posterior) mm (mean ± SD)	16.0 ± 7.7	13.5 ± 6.7	16.4 ± 7.8	0.005

Cystic content (n, %)				
Solid	166, 58.7	26, 65.0	140, 57.6	0.175
<50%	89, 31.4	8, 20.0	81, 33.3	
≥50%	28, 9.9	6, 15.0	22, 9.1	

Calcification (*n*, %)				
Non	260, 91.9	36, 90.0	224, 92.2	0.998
Macrocalcification	19, 6.7	3, 7.5	16, 6.6	
Microcalcification	4, 1.4	1, 2.5	3, 1.2	

Echogenicity (*n*, %)				
Hyperechoic	50, 17.7	6, 15.0	44, 18.1	0.669
Isoechoic	151, 53.4	22, 55.0	129, 53.1	
Hypoechoic	23, 8.1	5, 12.5	18, 7.4	
Heteroechoic	59, 20.8	7, 17.5	52, 21.4	
Nodule depth, mm (mean ± SD)	10.0 ± 6.6	12.1 ± 4.7	9.7 ± 6.8	<0.001
Time of surveillance, *m* (mean ± SD)	9.2 ± 4.7	10.4 ± 5.6	9.0 ± 4.5	0.090

**Table 2:** Correlation between nodule depth and nondiagnostic cytology (Bethesda 1) according to depth cut-off.

Nodule depth (mm)	n	Bethesda 1	Bethesda 2–6	*p*
<10	147	12, 30.0	135, 55.6	0.003
≥10	136	28, 70.0	108, 44.4	
<9	126	10, 25.0	116, 47.7	0.007
≥9	157	30, 75.0	127, 52.3	
<8	97	8, 20.0	89, 36.6	0.040
≥8	186	32, 80.0	154, 63.4	
act<7	67	5, 12.5	62, 25.5	0.073
≥7	216	35, 87.5	181, 74.5	
<6	55	5, 12.5	50, 20.6	0.232
≥6	228	35, 87.5	193, 79.4	

**Table 3:** Multiple logistic regression analysis for the correlation between nondiagnostic outcome and selected risk factors (OR and 95% CI).

Risk factors	Values	OR	95% CI	*p*
Gender	Female	Reference		
	Male	2.74	0.27–1.99	0.546
Age	Years	1.01	0.98–1.03	0.713
Nodule depth	<9 mm	Reference		
	≥9 mm	2.50	1.14–5.45	0.022
Dimension A	Mm	0.98	0.92–1.05	0.647
Dimension B	mm	0.96	0.89–1.03	0.258

OR- Odds Ratio, CI - confidence interval.

## Data Availability

The data used to support the findings of this study are included within the supplementary information file.
